# Human Development XII: A Theory for the Structure and Function of the Human Brain

**DOI:** 10.1100/tsw.2008.7

**Published:** 2008-07-13

**Authors:** Søren Ventegodt, Tyge Dahl Hermansen, Isack Kandel, Joav Merrick

**Affiliations:** ^1^ Quality of Life Research Center, Classensgade 11C, 1 sal, DK-2100 Copenhagen O, Denmark; ^2^ Research Clinic for Holistic Medicine, Copenhagen, Denmark; ^3^ Nordic School of Holistic Medicine, Copenhagen, Denmark; ^4^ Scandinavian Foundation for Holistic Medicine, Sandvika, Norway; ^5^ Interuniversity College, Graz, Austria; ^6^ Faculty of Social Sciences, Department of Behavioral Sciences, Ariel University Center, Samaria, Ariel, Israel; ^7^ National Institute of Child Health and Human Development, Jerusalem, Israel; ^8^ Office of the Medical Director, Division for Mental Retardation, Ministry of Social Affairs, Jerusalem, Israel; ^9^ Kentucky Children's Hospital, University of Kentucky, Lexington, USA

**Keywords:** holistic biology, theoretical biology, clinical holistic medicine, public health, neurobiology, brain, consciousness, mind, soul, true self, perception, will, human development

## Abstract

The human brain is probably the most complicated single structure in the biological universe. The cerebral cortex that is traditionally connected with consciousness is extremely complex. The brain contains approximately 1,000,000 km of nerve fibers, indicating its enormous complexity and which makes it difficult for scientists to reveal the function of the brain. In this paper, we propose a new model for brain functions, i.e., information-guided self-organization of neural patterns, where information is provided from the abstract wholeness of the biophysical system of an organism (often called the true self, or the “soul”). We present a number of arguments in favor of this model that provide self-conscious control over the thought process or cognition. Our arguments arise from analyzing experimental data from different research fields: histology, anatomy, electroencephalography (EEG), cerebral blood flow, neuropsychology, evolutionary studies, and mathematics. We criticize the popular network theories as the consequence of a simplistic, mechanical interpretation of reality (philosophical materialism) applied to the brain. We demonstrate how viewing brain functions as information-guided self-organization of neural patterns can explain the structure of conscious mentation; we seem to have a dual hierarchical representation in the cerebral cortex: one for sensation-perception and one for will-action. The model explains many of our unique mental abilities to think, memorize, associate, discriminate, and make abstractions. The presented model of the conscious brain also seems to be able to explain the function of the simpler brains, such as those of insects and hydra.

### INTRODUCTION

In our previous publications[1,2,3,4,5,6,7,8,9,10,11], we discussed the nature of biology, cell communication and deeply structured quantum fields, the structure of the neocortex, and human consciousness[12,13,14,15,16,17,18,19,20]. With such a background in the literature, you should be able to understand our motivation for the development of a new theory of the brain, as we need a strong scientific foundation for holistic medicine[21,22,23,24]. Holistic medicine can provide us with new treatments for many physical, mental, sexual, and existential human sufferings[25,26,27,28,29,30,31, 32,33,34,35,36,37,38,39,40,41,42,43,44,45,46,47,48,49,50,51,52,53,54,55,56,57,58,59,60]. Our former philosophical works on life, brain, consciousness, biological information, order, health, and disease have led to successful treatment of patients with many different health problems[61,62,63,64,65,66]. It is our hope that a more profound model of the human brain and consciousness can facilitate further progress. Our work is based on many publications by gifted researchers, such as Freud, Jung, Reich, Lowen, Rosen, Anand, and Antonovsky[67,68,69,70,71,72,73,74,75], and many others, who created the foundation for psychodynamic psychotherapy[76,77,78].

The present work is neurophilosophical, not traditional neuroscientific. It is based on many different sources of contemporary thoughts and is, thus, highly interdisciplinary. Our core ideas or *axioms* are:

1. Everything has a solid particle and an energetic wave aspect, according to the laws of quantum physics.
2. Everything is, thus, an aspect of energy. We live in a quantum world where everything, when it comes down to it, is made up of interfering, nonlocal energy fields of a quantum nature[8]. These fields are structured and can carry information that can be used by the living organisms[1,2,3,4,5,6,7,8,9,10].
3. Everything is, thus, an aspect of matter, such as atoms and molecules giving rise to biochemistry that can be used by the living organisms.
4. A living organism has two sides: subjective and objective; matter and consciousness that are bridged by biological mechanics, including – according to our understanding – both biochemical and informational quantum mechanics. Our hypothesis is that all organisms, even bacteria, have consciousness and a sense of subjectivity. We doubt that a virus has awareness or subjectivity, but then again, we need to define what we mean by "subjectivity" and "consciousness". They are two difficult and thorny terms with many meanings and connotations. *Subjectivity* is defined as the sense of a self, meaning being a "container" with consciousness of itself as being separate from the surrounding world. So, by *consciousness* we mean an entity's ability to represent, in its informational field, the inner and outer world in a meaningful way. Meaning rises from purpose or intent carried by the conscious entity; whenever something helps the being realize this inner purpose, meaning is felt. If a virus can sense its surrounding world without sheer mechanical stimuli, then it has awareness and consciousness by our definition. Thus, awareness is the subatomic ("quantum") quality derived from the entity's wholeness (the informational field) that "senses" (represents) the inner and outer world.
5. When two particles of any kind are contained in the same system, they share a common quantum state and, thus, stop being two separate entities. Their common quantum state can be used to coordinate the living system's parts with its wholeness, and this is true for life at all levels, from molecular ensembles, organelles, organelle-systems, cells, organs, organ-systems, organisms, and the levels of the outer ecosystem in which the organism participates. What is life in this sense? Is it not a supernatural vital force, a cause, an agent, or an independent being like the Indian or Christian concept of the soul? No, rather a materialization of an extremely complex and mysterious quality or aspect of the universal energy, providing the living being with autonomy, light, meaning, joy, purpose, will, and choice through billions of years of evolution.
6. The level of separateness defines the extent to which any part of a global ecosystem (living totality) has autonomy; the level of the organism has a rather high degree of autonomy giving rise to the organism's sense of self. Thus, every part of the whole has, to some extent, autonomy; parts that merge completely (i.e., cells that merge into muscle fibers) surrender autonomy to the system. All parts are partly autonomous and partly controlled by the levels above and below them.

7. We are well aware of the depths of the mathematical structures rising from chaos theory, fractal geometry, and complex dynamics[79,80], but we want to emphasize that we do not believe that self-organizing patterns can control either consciousness or behavior. Quite the contrary, we find the mathematical structures that are derived from the new mathematics[81] to be rather far from biological patterns, so it is quite obvious that we have not yet found the key to a profound scientific understanding of life.

This paper is our first presentation with interpretations based on these fundamental axioms of the current knowledge of the human brain; thus, we are taking an absolutely opposite viewpoint than normally done in neuroscience, where the nerve cell is seen as the mechanical unit of the brain, under control of genes, hormones, neurotransmitters, neuropeptides, and functional stimuli. Our hope is to be able to understand the nature and structure of consciousness and, especially, the functional relationship between consciousness, mind, and the physical (or cellular) body. Our hope is that such a model may allow us to understand emotions and psychosomatics, and, in the end, existential healing[23,24,26] and salutogenesis (defined by Aaron Antonovsky [1923–1994] as healing of physical and mental illnesses through the rehabilitation of "the sense of coherence"[74,75]).

The idea that the cell is conscious might seem strange to many people, but that was a conclusion reached by Sir Roger Penrose and other researchers at the SOL meeting at the Niels Bohr Institute in Copenhagen 1996[82]. Since this meeting, this has been our understanding of living organisms, i.e., that they always carry consciousness. This means that every cell in our body, to some modest extent, has an independent consciousness and subjectivity. It is this phenomenon that allows cells to develop into cancer cells and cells to be cultivated in the laboratory, also after the death of the multicellular organism. We know that this position will be hard to accept in modern neuroscience; that every nerve cell thinks for itself and makes its own independent decisions, which makes it so much more than just a small "mechanical computer". Sir Roger Penrose published a similar hypothesis in his book *Shadows of the Mind*[83]. It could be argued that we should draw a line somewhere between those unicellular organisms that may have primitive consciousness vs. the cells of our nails or hair[84], but we still find it most likely and in accordance with the philosophical principle of Occam's razor that consciousness is a trait carried by all living beings, but on different levels of complexity.

### DOES A ZYGOTE HAVE A SENSE OF SELF?

This leaves us to the natural question, Does a zygote have a sense of self and consciousness? Based on empirical research with people who re-experience their conception, the answer definitely seems to be affirmative, but the objectivity of such studies has been disputed[84]. If you think this is a little too far-fetched, we must tell you that in spite of being quite skeptical ourselves, we as scientists often see patients spontaneously regress all the way into the womb in intensive holistic therapy. The first author has, after 20 years of consecutive therapy, suddenly regressed all the way back to the zygotic state during holotropic breastwork[86]; according to this experience, there was indeed a conscious "I" from the very beginning of life[87], but then again, when you become very experienced late in life, you start to relax your skepticism a little and believe in all sorts of things, and then you are not really reliable any more, are you? To keep it simple, which is necessary in order to comply with Occam's razor, the sense of self (being an independent soul) does not develop gradually from the zygote – embryo – fetus – newborn – infant – child – adult – old age; this feeling of being an autonomous creature is with us from the very beginning as an innate trait of the person. What is developed through time and experience is the level of complexity of the mind (brain-mind), and perhaps also the complexity of the being.

Many scientists believe that the human brain is a kind of independent computer, able to understand almost everything in this universe. However, at the same time, it seems clear that we human beings will never be able to comprehend fully consciousness and the brain itself. This paradox may be a consequence of a narrow, rationalistic, and materialistic interpretation of reality[1,2,3,4,5,6,7,8,9,10].

If the interpretation of reality gets more complex and less naïve, this might give us a chance to develop a more transcendent and deeper understanding of the brain and consciousness. The description of life as a complex, dynamic, and information-directed self-organizing system[4] is an example of this point of view, and it can settle the above-mentioned paradox. From this perspective, consciousness is seen as a characteristic of all living things. Because of the relative high degree of outer separateness, self-consciousness is especially prevalent in the human brain, which has a large degree of autonomy; the abilities of the human mental self (Ego) are complex and advanced. The body is the next fairly independent biological entity, carrying, as Freud noticed correctly, the consciousness of the Id (and the body-mind). Finally, the human wholeness also carries its representation of us called our "spiritual self", "true self", or "soul". This gives man three dominant representations of self: the wholeness-related self (below called the Soul), the brain-related self (below called the Ego), and the body-related self (below called the Id). Then we have the "I", the integrative self, that emerges from an organic synthesis of body, mind, and spirit. This "I" is often in spiritual literature and poetry called the "heart" (compare the lyrics of Madonna: "When your heart is open"). In Freud's work, the superego is synonymous with the soul; Freud often said that everything good in man comes from the superego[67].

The interesting question, "Doesn't the self operate through the brain?", leads us to an immediate "of course" and a secondary remorse on deeper reflection that, in the end, brings the realization that our thoughts and behavior might be controlled by our self, but feelings, sexuality, and spirituality are as well controlled by our self, so are we really living in the brain? This is most definitely not our experience, if we must be honest. Emotions are felt in the body and our sexual feelings are definitely felt primarily in our sexual organs, and maybe this is not merely a joke, but a human biological reality that must be respected, as Freud insisted.

We know that there seems to be an interpretation of our feelings in the limbic system and without this happening, we could not be mentally aware that we have been emotionally hurt or sexually excited. However, to insist that feelings and being is merely a brain thing is to insist on something that obviously conflicts with our common sense (senses communis). As therapists, we are emotionally oriented people and we really like to place the "I" in our hearts much more than in our brains. This gives a much more human contact and a much richer emotional life. On the other hand, we must agree that mental consciousness obviously is focused in the middle of the brain (in what the Indians have called the "3rd eye" for 7,000 years). We like to use our experience as a basis for understanding, not vice versa, and this is the true reason for making this paper: We need to stop thinking so much, and start to sense and explore our inner self in order to really understand what is going on. We suspect that many neuroscientists often miss the obvious truth because they do not sincerely feel what is happening inside and reflect on it. Basically, this is a question of using subjectivity in research and putting sufficient emphasis on the qualitative aspect of science. In this paper, we want to analyze the implications of this for our understanding of the brain.

### CONSCIOUSNESS

All the cells of the organism carry consciousness. Human consciousness is basically embedded in a quantum field that arises from the combination of all individual cells' consciousnesses[4,5,6,7,8,9,83]. This is highly debatable, we must admit, but we humbly ask you to play with this idea before you decide to dismiss it. This structure of Soul, Ego, and Id, all carrying semi-individual consciousnesses, means that the whole organism (the "heart" or "I") can "see" and analyze all its lower-level conscious functions; thus, both brain-mind functions, body-mind, and even wholeness-related "spiritual intelligence" functions are observed and interacted with by the "I". It is very usual in therapy that a person suddenly observes an inner process of cancer or observes his own autistic side, and this could not be done without the ability of such a “meta-perspective” provided by the abstract “I”. This is somewhat related to the strange observations that patients have during surgery where they can observe the surgical theater from above, from a viewpoint where they obviously feel out of their body. Most interesting, this shift of perspective also often happens in intensive holistic therapy, so it is hard for us, as therapists, not to believe witnessing this happen all the time. Not that we understand it, or even like it. Human consciousness is way too mysterious to be cozy.

The “I” or “heart” can see the brain from a perspective “outside” the brain, but inside the organism, and the body can see the brain and the brain can see the body – energetically, or if you prefer, by direct transference of information from one “wholeness” to the other. Is the wholeness of the body different from the wholeness of the brain? Does an organism have one wholeness? Does an organism behave as one entity? The unreflected answer is that the organism carries the wholeness and its organs do not. However, after deeper reflection and meditation on ants and corals, and after that on human societies and the way consciousness is manipulated in a society collectively, i.e., by the media, we must admit that the organisms are not that free to think, feel, and act as we would like them to be. After thorough studies on sexuality and human unconsciousness (unintegrated traits of brain-mind and body-mind), we must admit that our organs are pretty powerful actors in their own right. So the picture is much more complicated and, in the end, every level of us has some degree of dependence and freedom.

The “I” can use the body’s emotional intelligence – the faculty of intelligence connected to the body-cells’ collective consciousness field (and, of course, the wisdom of a mature brain’s corticolimbic system) – to get a clear picture of the mental processes and the basic machinery that creates its own mind. This is the typical perspective of the Tibetan Buddhists Yogis, reached in deep meditation, but only little acknowledged in the West until recently[87]. You could argue that these are subjective reports of their mental states and experiences, and, thus, they are probably not good arguments in support of our thesis in a scientific journal. However, our direct experiences of the world might be as real as our mental reflections on it; we would actually argue… more real!

The brain is represented in the organism’s wholeness in the same way as the organism is represented in the brain. You could argue that you have never seen your own brain, but if you can sense where your mental activity is centered – right behind your eyes – then your wholeness has already acknowledged that you can sense your own brain, or at least its energy and quality. So what we basically claim is that the brain cannot be understood without understanding the “I” and its consciousness, and Id, and the Soul, and their individual consciousnesses.

In a well-integrated organism, the self rules (“I” am in power); from its placement at the top level of the organism, it can strongly impact what is going on at all the lower levels, including in the brain. The model we are going to develop further was originally made by the psychoanalysts to allow us to understand how I-born consciousness (as in “living by heart”) can be causal in our life[67,68,669,70,71,72]. Interestingly, this model seems to be the normal understanding of man in most premodern societies[9] and, most interestingly, opens up to an explanation of collective consciousness[9,68,69] that is normal in premodern societies, but almost forgotten or neglected in our culture, in spite of Jung[68,69], Grof[85,86], and other prominent psychic researchers who stress its meaning and importance to us. It seems that it actually came from the premodern cultures into psychoanalysis, especially by Jung. The model we present is not purely based on theory, but on much practical experience, and it is also in accordance with the philosophy of life that has arisen from our research in quality of life and health during the last 2 decades.

### UNDERSTANDING THE COMPLEX PATTERNS OF BRAIN ACTIVITY

To understand the various functions of the brain, we need an integrative theory for brain function that accounts for the control of the mental functions on the highest level of the brain. The multidimensional connectivity that follows the extreme con- and divergence in the architecture of the human neocortex, the results of countless EEG measurements, and the measurements of the high and almost constant brain energy usage, indicates that the cortex cerebri is a machine that almost continuously delivers a huge selection of patterns that float into each other[79,80,88,89,90,91]. Mathematical analyses[90,91,92] have indicated that the cerebral cortex cannot organize the patterns by itself in a meaningful way, leaving us with the most fundamental problem of how the brain is controlled.

It may be assumed that sensory inputs to the brain temporarily can stabilize its chaotic, neural, self-organizing patterns, creating a sensory perception of simple information-directed self-organization. However, this does not explain much – thinking, understanding, perceiving, etc. Therefore, another much more efficient and innate organizing system may exist that makes the brain function as it does. Morphological and evolutionary data[93,94] seem to show that the nervous system is developed and functions through an intense communication with self – or in more scientific language, the brain is totally imbedded in the complex informational dynamics of the organismic wholeness. The organismic wholeness entirely depends on a well functioning, living brain as expressed by John Zachary Young (1907–1997), professor of anatomy at the University College London: “No brain, no mind, no nothing.”

Actually, the brain is completely absorbed in the organism and has, as we see it, no completely independent function of its own, in spite of it being, in many structural and physiological ways, a highly autonomous organ. Even the slightest action on the brain is, in some way, influenced by the totality and intentions of the being who owns it.

One can ask, At what stage of embryological development of an organism, like a bacterium or a human being, is there a communication between the brain and self? We know little about how bacteria process information, but they obviously do, and the close distance between the genes and their global level tells us that there must be an intense inner representation of the bacteria’s wholeness. Do we have a self when we are at the single-cell level of development viz. the zygote? Yes, we seemingly do. Do we have self even before conception? No, at least not in our philosophy. Then again, it is wise to remember that half the population of the planet would disagree here.

In neuroscience, it is normally believed that a lot of the cortical-subcortical activity is spontaneous, involuntary, automatic, and subconscious. This is often presented as a fact in many standard textbooks of neurobiology, neuroscience, and neurophysiology, but it is worthwhile to remember that we never have seen a brain keeping these “automatic” functions on its own (*in vitro*), and if we count the consciousness of the self and the body, this might not be the case at all. The interesting thought-experiment to do here is to imagine a person’s brain isolated and fed in a jar: Will this brain still be able to think and feel like the person it came from? Will it still function at all? We know, of course, that *in vitro*–developing neurons still fire, but will this activity be able to create any collective meaning without the informational guidance from the self and the body? The consequence of our thinking is that this is not possible. Unfortunately, we do not know of any experiments that can decide this for us.

Contrarily, we know of many experiments that indicate that the functional order of the brain is highly fluent and rapidly reorganized. The cortical representation of all sensory and motor functions in neural maps and their well-known and quite mysterious, momentary reorganization[88] seems to confirm that brain function is controlled from “a level above” because the representations are not fixed in the physical brain – the maps are not hard wired. Studies of blood flow and lesions show a hierarchically ordered structure of representations in cortical networks[88,93,94]. These examples are in accordance with the psychological developmental studies showing that the consciousness has its starting point in the functions of the body, with the primary sensory and motor areas creating the foundation for the hierarchy. The higher integrative areas create the intermediate, and the highest integrative areas – especially the prefrontal cortex – create the top of the hierarchy[88]. It is well known that some brain lesions, like that of Broca’s area, are followed by expressive aphasia. So there are also many hard-wired solutions in the brain, of course, on lower levels of the brain, but all high-level, consciousness-related organization seems to be informationally directed and we believe that the self provides this information, and that the self thus controls its brain. This link between self and brain is crucial for our understanding of, for example, the altered brain function and physiology in illnesses like depression and schizophrenia.

In Appendix 1, we give a short evaluation of the value of central experimental data for our understanding of the brain, with special attention on the function of the cortex cerebri. We will now discuss how the brain functions according to our holistic understanding. The fundamental structure of the neocortex[95,96] was reviewed in Hermansen et al.[11] and will not be repeated here.

### A THEORY FOR BRAIN FUNCTION

What does the brain do? Basically, the brain connects Ego, I, Soul, and Id to the outer world. It carries the organism's rational interpretation of the world and allows it to realize the intentions of self and Id through plans carried out in time and space. The brain creates mental perception, rational understanding, and visual, auditory, somatosensory interpretation of the inner and outer world, and proper rational actions and inner adjustments from the Ego's, Soul's, Id's, and I's intent.

One could ask if I, Soul, Ego, and Id are generated from a well-functioning brain, and this could very well be so. The different inner personalities that we label all these names could easily rise from a less-than-perfect integrity of the brain. So, one cannot judge just by thinking, if there is any rationale behind the more complex model of human reality that we propose; we do it because we respect our "common sense", our direct experience of life; it is this sense that allows us to help our patients in holistic therapy, and provides us with the power of healing. So, we cannot just give it up, and when we take our experience to meet neuroscience, things do not fit. What we feel and experience is simply not compatible with the mechanical interpretation of reality we find in contemporary neurophysiology books; even profound books, like *Principles of Neural Science*[88], do not reflect on the quality of existential depth, joy, light, and innate wisdom that we sense is connected to consciousness. Frankly, the concept of consciousness is hardly addressed in neurophysiology of today. The "hard" problem (how subjective consciousness is produced from chemistry and physics) has not been solved and is, much too often, just ignored.

This is done through intensive mapping of the inner and outer world. The brain is in touch with the outside world through the senses and the apparatus of movement, and in touch with the person's "inner world" through feelings, intuitions, finer sensations, intentions, states of consciousness, and being; dreams are very much the materialization of this inward contact.

Throughout life, a more and more detailed model of reality is built up, using the fundamental dimensions of space and time. The brain and mind harvest experiences through the presence of sensory qualitative units called "qualia" – like the color red – that is solely produced by the nervous system and the organism itself; a nervous signal cannot, in principle, be read (unless you accept that it can carry a more subtle and finer level of information, i.e., quantum level information). Qualia are combined through time and space into elements that can be perceived and manipulated, giving birth to the phenomenological world. The intensity of qualia is established, and its location in time and space is noticed, and all these neural measurements are integrated into a dynamic perception. Mental elements can be static or dynamic, corresponding to nouns and verbs in language (see Chomsky's famous concept of "deep structures" of language, and Piaget's model of development of human consciousness[97,98,99]). The nonverbal mental phenomena, such as pure visual images, touch, taste, and emotions, are also based on qualia, but they are often just taken for granted and not abstracted to higher logical levels. However, they can be, as in the Indian and Tibetan art of erotic tantra, where sexual feelings and unified sexual poles are abstracted to oneness ("sunya")[73].

The mind is, thus, basically nothing but a highly dynamic model of reality constructed in this way, just by combining the elements on higher and higher, more and more abstract levels through experience and memory, and the mental faculty of abstraction and concretization. The model is organized through association and dissociation. Logic and sets are used for giving rational structure.

The brain is constantly preparing behavioral and perceptual strategies to meet the intentions of the whole organism (the "I" motivated by "Soul, Id, and Ego" according to psychodynamic theory). The brain interprets all experiences and sensory inputs from the outer world in agreement with the organism's intentions, accumulating concrete strategies for action and for perceptual and intellectual analysis. These strategies are gradually revised as new goals emerge through a changing life. When the resistance is too big – when realizing one's dreams and intentions is too difficult and painful – the goals are replaced, in resignation, with smaller, more obtainable goals. Such events result in the degeneration of intent (life mission[12,13,14,15,16,17,18,19,20,50,51,54,55,56,57]) and personal character, which sometimes even leads to mental illness like depression and schizophrenia, as suggested by Bleuler, Freud, Jung, and others.

The only interpretation of the brain that is in accordance with all the collected data from all the sources mentioned above is that the brain is a pattern machine that continuously produces concrete and abstract patterns combining into "sensory motor pictures". It seems that this process is guided by the organism's abstract high-level perceptual faculty of finding meaning in chaos and a similarly abstract faculty of intent. It seems that it is the self (the organism's wholeness) that guides the brain in its ability to make plans for achievement and realization of the abstract and concrete goals of the human being. The reality is interpreted in agreement with the intention and is represented for the conscious wholeness, where it is evaluated and processed. This seems to be in accordance with Arthur Schopenhauer (1788–1860), a German philosopher who believed that the will to live is the fundamental reality.

### INFORMATION-GUIDED SELF-ORGANIZATION OF THE NEURAL PATTERNS

We have analyzed the process of morphogenesis and found that it happens through information-directed self-organization of cells and tissue; all cell movements and differentiations are initiated and directed through information-directed self-organization of molecules and organelles[4]. It seems reasonable to suggest that the brain functions in a completely similar way through information-directed self-organization of complex, dynamic, hierarchically organized, neural patterns.

The neural connectivity patterns are specified through information-transferring interactions on many levels of the living organism. This means that both the patterns of connectivity and the functional neural patterns of the working brain interact with the information-bearing, complex, dynamic processes of the biological system; please recall that we found this to be a real phenomenon existing in the organism at a quantum level[8]. The functional neural patterns are different from the structural, but when structures and functions are developed in parallel through evolution, it must be that the functional patterns also interact with the informational level of the organism. A fine example of this is the *Hydra*; in the Hydra, the neural network is constantly updated by the organism. If the body of hydra is reshaped by cutting, the neural information reconstructs it with no hesitation.

We can look and make sense of even the most complicated of patterns, such as turbulent water flowing, growing plants and ecosystems, or computer-produced fractals. From this it is clear that our brain has the capability to form extremely complex patterns for perceptual use – much more complex and complicated than those structures that are before our eyes. The brain formats extremely complex patterns. This neural "modeling medium" (or matrix) can be organized either by sensory input or by the consciousness and intention of the organism.

Since our senses are always flooded by information, it is obvious that a considerable and continuous selection of the incoming data is happening at all times. The intent of the self determines what is interesting for the being from an existential perspective and, therefore, defines the contents of its perception. Therefore, it is correct to state that the content of consciousness is actually caused by the self. This is an extremely interesting conclusion as it makes the perception of the mentally ill, including delusions, hallucinations, and emotional flattening, understandable[17,50,51]. Most interestingly, intent is connected to the philosophy of life, and revising one's philosophy of life seems to alter the realm of perceptions completely; the patient in deep philosophical exploration is often "traveling from heaven to hell and vice versa". Also, the perception of body, sexuality, the partner, etc. is completely moldable by the person's philosophy of life.

The huge mass of data not found to be relevant for the realization of the organism's intentions is selected by the materialization of a "pyramid of consciousness" from the abstract intentions to the concrete perceptions and behaviors; the intentions materialize "concrete plans" that support the specific sense impressions. For the organism's wholeness, intention materializes existentially relevant experiences; in the brain, intentions materialize the physical and well-known description of the world used for everyday living, i.e., the interpretation of the reality.

On a mechanistic, informational level, intentions must come to the brain in the form of superior matrices of guiding patterns – these patterns correspond to those of the organism's wholeness; they organize the top-level patterns selected by the functioning brain and the brain's self-organizing nature takes care of the rest. The organism's plans and strategies for self-realization are carried out in agreement with the rational interpretation of the organism's sense impressions. In humans, we know that dreams are very important in this process.

### A DOUBLE HIERARCHICAL MODEL FOR THE REALITY REPRESENTATION OF CORTEX CEREBRI

In order to simplify this description, we will only concentrate on the largest structures of the prosencephalon. The subject for the discussion will be the integrative structures, such as the limbic system of cortex cerebri, the basal ganglia, and thalamus. The last two of these areas will be considered as equally connected with the areas of cortex cerebri. We think these structures represent a person's "emotionally close social relationships in the group" (the limbic system), groupings in the basal ganglia corresponding to the motor and verbal activities related to the outside world, and the whole perceived and cognized reality model in the thalamus and the related cortex. In the following, these structures are included in the term cortex cerebri.

These two hierarchies, organizing the two fundamental abilities of the brain, are both coherent with the reality outside the brain. The structure of the hierarchies is a complex "Chinese-box-type" system (see Fig. 1), based on the fractal informational system seemingly existing in all biological systems[6,7,8,9,10]. If the cerebral cortex is seen as one whole organ, its parts, or building blocks, will be the functionally defined areas, such as Broca's area, known as Brodmann's areas. These again are separated into supracellular structures, e.g., feature detectors in the visual cortex, which again are built up by cells, etc. as discussed above. The second hierarchy, roughly speaking, goes lengthwise through the brain. The primary sensory input from vision, hearing, and somatic senses, gives representations of these sense spaces, in particular places. Seemingly, these areas converge to superior integrative areas that again converge to the highest integrative areas. The last ones are directly related to motor cortex, from where movement is controlled. However, the somatosensory cortex is also placed next to the somatomotor cortex. Fig. 2 shows these hierarchies separated and together.

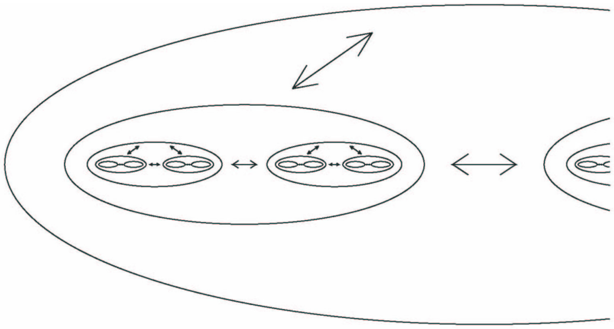

**FIGURE 1.** The living organism seen as a fractal structure with interacting parts on many levels. The arrows illustrate the information-transmitting interactions and are based on generalized empiric results. The figure illustrates how biologic informational systems are structured as a fractal "Chinese box"; the information-transmitting interactions are seen between the different parts of the same level and between levels.

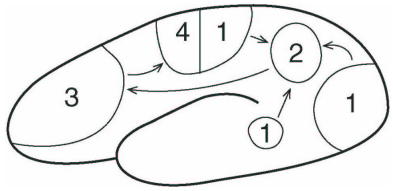

**A**

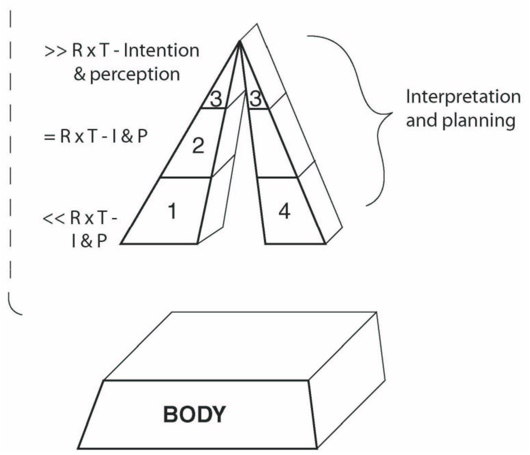

**B**

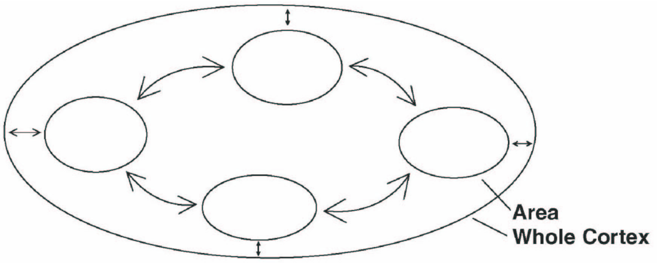

**C**

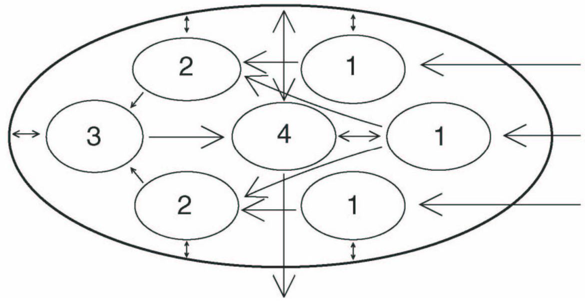

**D**

**FIGURE 2.** A representation of the model of reality in the (human) brain as a consequence of a double hierarchy. (A) Parts of the brain that interact by each other. (B) The hierarchy of quantified qualia organized in space and time (I = Intention, P = Perception). (C) The Chinese box hierarchy of interactions in the brain. (D) The double hierarchical representation of reality in the brain.

### EXPLANATION OF THE ABILITY TO ASSOCIATE, DISCRIMINATE, AND ABSTRACT

The cerebral areas represent a separate level and use a time-space hierarchy (see discussion above), but they also have to interact with the superior wholeness of the brain. Data corresponding to everything that has happened on each level could possibly be stored in each of the cortical areas from the complex dynamic at the lower levels. Recall that the storage of data may happen in an extremely controlled way because each area contains an unimaginable amount of information.

A consequence of the ongoing information-transmitting interactions on all levels of the brain is that every recall of data happens in an associated way. This means that only the data that pass into the larger pattern are retrieved and it is the most superior pattern that organizes the complete recall, which in this connection is the intention. The ability to discriminate is presumably achieved through the structure of the functional patterns themselves. The ability to abstract and generalize follows from a corepresentation of many elements having common traits in an n-dimensional, self-organizing, associative room (see Ventegodt et al.[7] for a study in this geometry), which will make up an informational body that forms exactly as the generalization or abstraction of all represented elements in this area. Such bodies (or sets) that are built by big amounts of smaller bodies will correspond to higher levels of abstraction in a similar way.

### A PROPOSAL FOR THE GENERATION OF THE REALITY MODEL IN THE BRAIN

Fig. 3 shows how a four-level model of reality can be created from simple repetitive cogroupings of elements of quantified qualia in space and time. First, sense impressions are analyzed to meaning units in meaning unit analyzers (this may be too speculative, like the “grandmother” cell, and the mechanism might be energetic and not mechanic at all; compare the visual feature detectors). The distribution of these “meaning units” in space and time is determined in the representation as positional information (compare the traditional use of this concept in ontogenesis[see 5]) – the quantifying of the qualities. To realize the relationship between space and time in these groupings at different levels must be imagined. Thus, the fundamental structure of space-time must be fully understood for us to comprehend consciousness fully.

The first grouping level causes the creation of sense elements corresponding to the perceptual level of qualia localized in space and time (this process takes milliseconds). On the motor side, a corresponding grouping to motor elements exists. The second grouping level is a cogrouping of the different sense modalities with sense elements in bigger space and time (many milliseconds or seconds) to perceptions. These cover the whole sensory-perceptual space. In the same way, motor elements are grouped together to create concrete movements.

The third grouping level is higher space and time (seconds or minutes) groupings of perceptions and behavior. This gives a reason-effect, with relationships between perceptions and behavior as possibilities, and those between behavior and perceptions as consequences. In the last case, the possibilities of a perception for action, and actions for perceptual consequences, is getting clear. On the third level, the cogrouping of the perceptions with behavior in space and time gives the reality models concrete functional elements, e.g., as a cup, together with those processes that move and transform the elements into each other (the cup, for example, can break into pieces when it is dropped on the floor). A child’s limited intellectuality, and its ability to interpret the concrete objects and possibilities of the surrounding world, has been built up at this point. Language, with its nouns and verbs, is introduced at this stage of life. Here, it is central to understand that the reality of the child is developed in identity with biological intentions and needs.

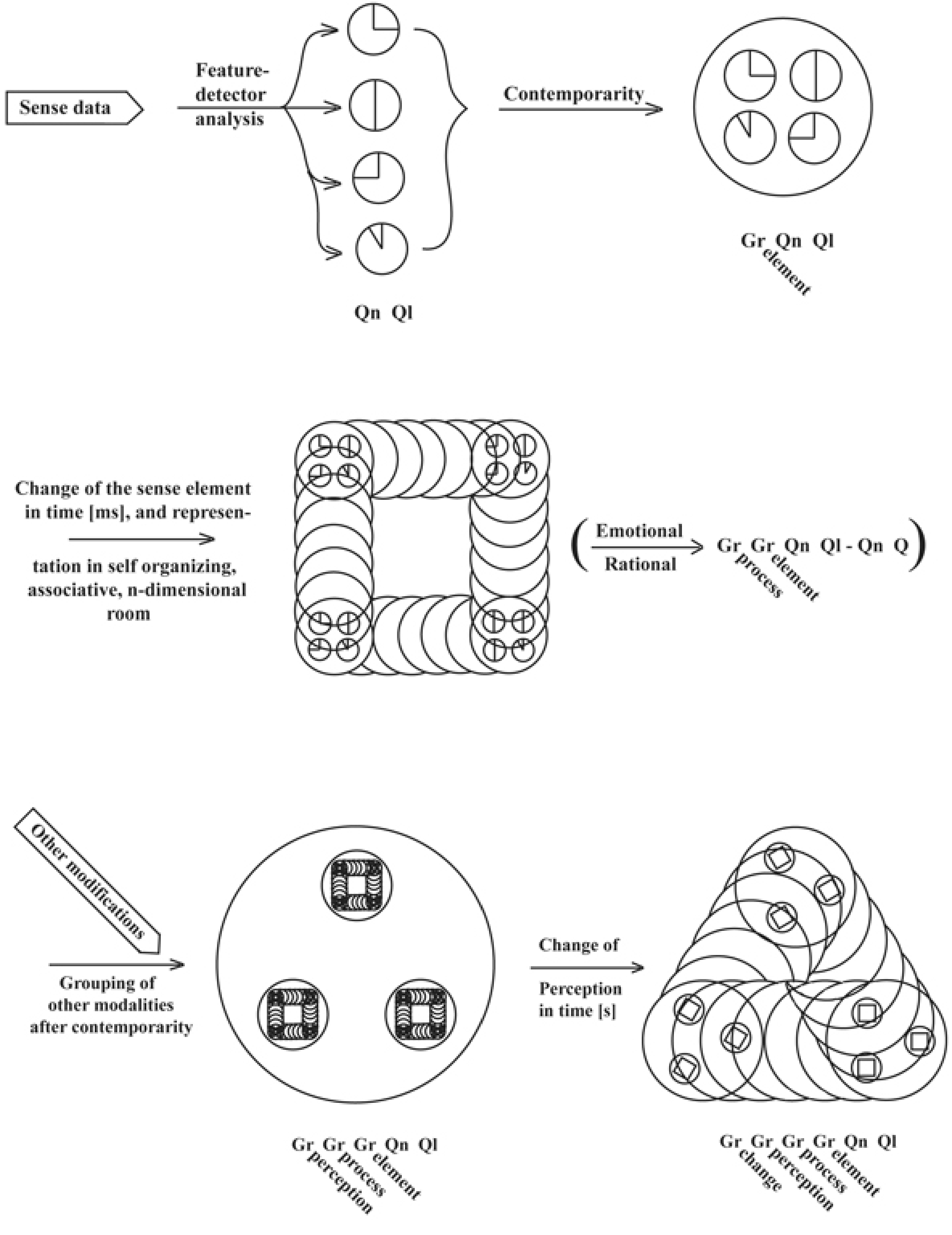

**FIGURE 3.** A model of the representation of reality in the human brain (see text). Continues next page.

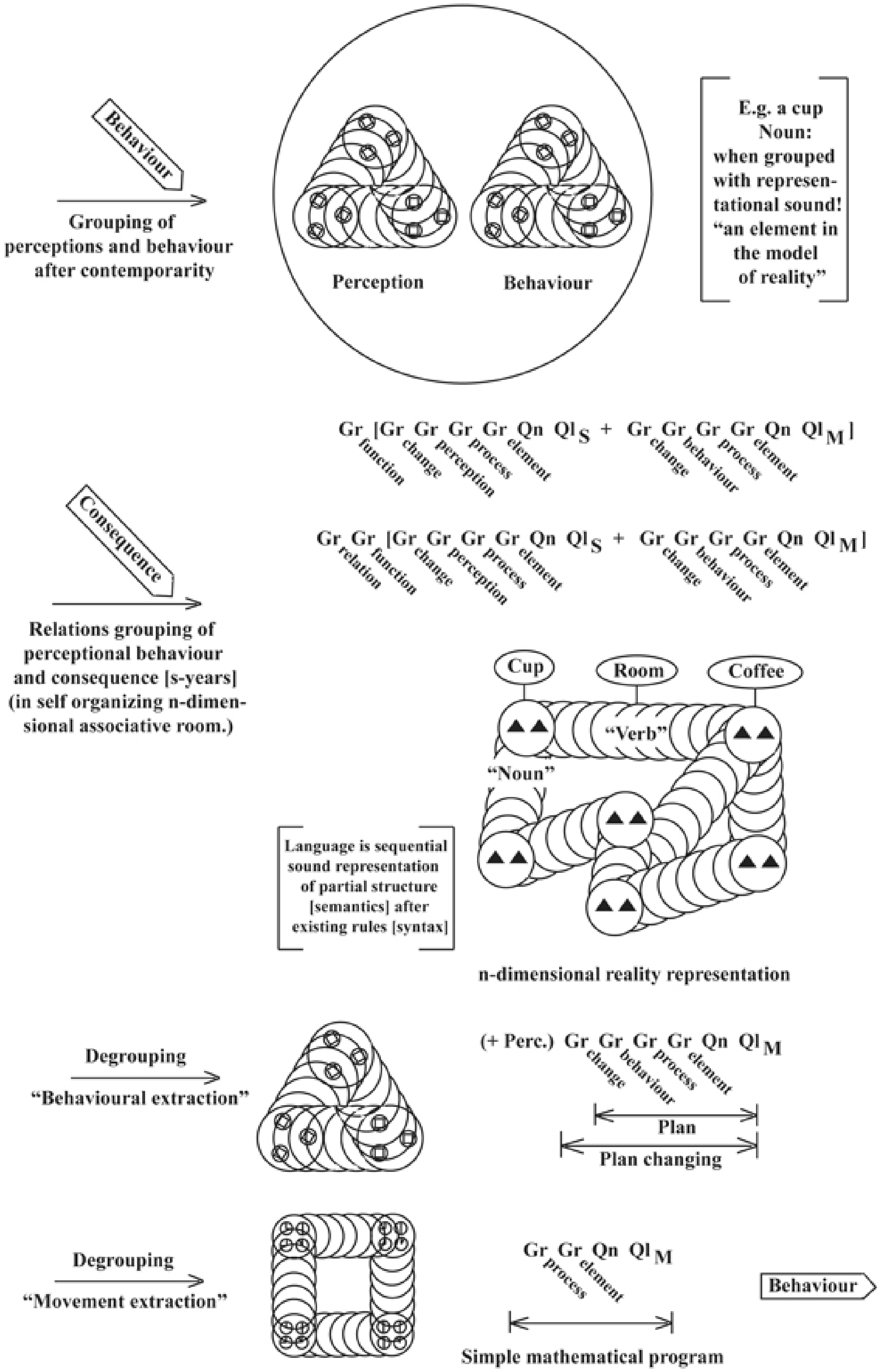

**FIGURE 3 continued.**

The fourth group level replies to the groupings of possibilities and consequences, on higher space-time levels (from minutes to years) that fit with the more complicated intentions of human beings. In the reasonability of interpretation, this corresponds to intellectual development because the reality interpretation here is lifted from the concrete world into the world of ideas, abstractions, principles, hypotheses, lawfulness, and logic. In the emotional reality interpretation, this corresponds to the recognition of extremely complicated situations in the reality, outside the body.

The hierarchical representation through cortex cerebri fits with the existing data of the brain. This representation also makes good sense, since this fits with cogroupings of sense data and data concerning motor functions, in a number of levels that, in the end, represents human conceptions, ideas, and intentions.

The purpose of the first concrete grouping levels is to realize the concrete intentions of the child as eating, drinking, playing, etc. On the other hand, the abstract groupings serve the purpose of realizing the long-term intentions of the adult individual, through huge spaces and times.

For the brain, it is the case that the intentions are superior patterns that organize the highest level of cogrouping of the elements of the reality model. A specific intention, in this way, results in a selection of specific superior consequence groupings, and these again correspond to a plan or strategy. This strategy is realized through a degrouping to sensory motor elements, completely analogous to the superstructure of the reality model, see Fig. 3.

The intention, therefore, is the innermost craft that organizes the reality model in the brain. However, the coordinated learning acts directly on the intention. Also, a great part of the parent's reality interpretation is transferred, in a direct way, through information-directed interactions to the child as learned ideas with value ladings attached to these. Therefore, the reality model is often filled with glaring conflicts, misty ideas, and direct contradictions. This results in a really complicated structure caused by the demands of social interactions.

### DISCUSSION

We do not believe that the brain is a "neural network" that has a conscious activity of its own; neither do we believe that such an "isolated brain" could have independent activity corresponding to thoughts and dreams. We believe that models for self-organizing associative memory have demonstrated a fundamental disability in thinking and perceiving; as the computerized "neural network" of an isolated brain cannot have independent activity like thinking. We believe the brain to be a highly complex pattern machine that continuously produces concrete and abstract "sensory motor pictures" guided by the intention of the wholeness. The mechanism by which this is done by the brain is by using information-directed dissipative neural patterns, the information coming from both the senses and the organism's wholeness. The brain's central job is to make meaningful plans for achievement and realization of goals presented to the brain at its top level by the human wholeness (the "I"), often in dreams. The reality is interpreted in agreement with the intention and is through the brain's highest level represented for the organism's wholeness in which it is evaluated emotionally.

The presented model is built on a number of axiomatic statements derived from the former papers in this series. It is quite surprising to us that it is possible to get such a clear picture of human brain function that is helpful in so many ways, i.e., in explaining the perceptive distortions of the mentally ill from the degeneration of intent. The model seems highly helpful in relation to clinical holistic therapy, where these distorted perceptions in the form of transferences and projections are happening at all times. The clear understanding of their neural basis will presumably make it much easier to deal with the mentally ill in therapy, and increase the number that can be helped by scientific holistic medicine. The problem of the proposed model is that you need a holistic philosophy, admitting the individual cell consciousness, etc. to use it.

### CONCLUSIONS

We propose a theory for the function of the (human) brain, claiming that it works on a mechanical level through information-directed self-organization of neurally produced, extremely complex patterns, which only add up to meaningful perceptions and actions because of the fundamental will, or intentions, of the individual. We likewise assume that the morphogenesis of the brain happens through the information-directed self-organization of cells and tissues, and that this informational link is active throughout the individual's life, securing an extremely close informational connection between mind and self (the wholeness of the organism). All growth, absorption, and modifications of nerve cells, axons, and dendrites are guided through information-directed self-organization of the molecules and organelles[1,2,3,4,5,6,7,8,9,10].

When we look at the most complicated visual patterns, e.g., turbulent water flow, growing plants, or computer-produced fractals[101], and understand how fast and direct the brain interprets even the most complicated of visions, we find it obvious that a "patterned medium" exists in our brain that immediately forms even extremely complex patterns; these extremely complex patterns are organized in many hierarchical levels to create the well-known model of reality in the human brain.

A possible consequence of the information-transmitting biological interactions in the "deep quantum field"[8] is that recall of information happens in an associative way. Only memorized patterns that resonate with actually activated patterns are recalled. This ability to discriminate is achieved through the structure of the functional patterns themselves. The ability to abstract and generalize follows from a corepresentation of many elements having common traits in an n-dimensional, self-organizing, associative space.

The cerebral cortex thus embraces two functionally separate but closely interrelated informational hierarchies, one for sensing, perceiving and interpreting, and another for intending, planning and acting. One of these is a variant of the Chinese box system. In this, the brain is built by structures in the cortical areas separated in supracellular structures that, in turn, are built by cells. In the second hierarchy, the primary sensory input from vision, hearing, and somatic feeling gives representations of these sensory spaces. The last hierarchy is directly related to motor cortex, the part of the brain from where movement is controlled.

This sums up our proposal for the generation of the model of reality in the (human) brain. The many-leveled, organizing, biological (positional) information organizes the distribution of the qualia (meaningful units) in space and time. We suggest that the intentions of the whole individual are represented in the brain as superior patterns that organize the highest levels of the model of reality in the brain; this high-level cogrouping of all the elements of consciousness by the intention is the innermost craft that organizes the perceptual and acting powers of the brain. The brain is structured by evolution to transform all modalities of sensation and intention into its integrated perception of reality, in a form that empowers it for optimal action to achieve self-actualization – the full realization of the abstract "I".

### ACKNOWLEDGMENTS

These studies were supported by grants from IMK Almene Fond. Our research in quality of life has been approved by the Copenhagen Scientific Ethical Committee under number (KF)V.100.2123/91. We are grateful to professor Hans Hultborn for his review and comments to the first version of this paper, and Vinod D. Deshmukh for his thorough critique of the third version.

## References

1. Hermansen, T.D., Ventegodt, S., Rald, E., Clausen, B., Nielsen, M.L., and Merrick, J. (2006) Human development I: twenty fundamental problems of biology, medicine, and neuro-psychology related to biological information.

*TheScientificWorldJOURNAL* **6**, 747–759.

2. Ventegodt, S., Hermansen, T.D., Nielsen, M.L., Clausen, B., and Merrick, J. (2006) Human development II: we need an integrated theory for matter, life and consciousness to understand life and healing. *TheScientificWorldJOURNAL* **6**, 760–766.
3. Ventegodt, S., Hermansen, T.D., Rald, E., Flensborg-Madsen, T., Nielsen, M.L., Clausen, B., and Merrick, J. (2006).Human development III: bridging brain-mind and body-mind. introduction to "deep" (fractal, poly-ray) cosmology. *TheScientificWorldJOURNAL* **6**,767–776.
4. Ventegodt, S., Hermansen, T.D., Flensborg-Madsen, T., Nielsen, M.L., Clausen, B., and Merrick J.(2006). Human development IV: the living cell has information-directed self-organisation. *TheScientificWorldJOURNAL* **6**, 1132–1138.
5. Ventegodt, S., Hermansen, T.D., Flensborg-Madsen, T., Nielsen, M.L., Clausen, B., and Merrick, J. (2006) Human development V: biochemistry unable to explain the emergence of biological form (morphogenesis) and therefore a new principle as source of biological information is needed. *TheScientificWorldJOURNAL*. **6**, 1359–1367.
6. Ventegodt, S., Hermansen, T.D., Flensborg-Madsen, T., Nielsen, M., and Merrick, J. (2006). Human development VI: supracellular morphogenesis. The origin of biological and cellular order. *TheScientificWorldJOURNAL* **6**, 1424–1433.
7. Ventegodt, S., Hermansen, T.D., Flensborg-Madsen, T., Rald, E., Nielsen, M.L., and Merrick J. (2006) Human development VII: a spiral fractal model of fine structure of physical energy could explain central aspects of biological information, biological organization and biological creativity. *TheScientificWorldJOURNAL* **6**, 1434–1440.
8. Ventegodt, S., Hermansen, T.D., Flensborg-Madsen, T., Nielsen, M.L., and Merrick J. (2006) Human development VIII: a theory of "deep" quantum chemistry and cell consciousness: quantum chemistry controls genes and biochemistry to give cells and higher organisms consciousness and complex behavior. *TheScientificWorldJOURNAL* **6**, 1441–1453.
9. Ventegodt, S., Hermansen, T.D., Flensborg-Madsen, T., Rald, E., Nielsen, M.L., and Merrick, J. (2006) Human development IX: a model of the wholeness of man, his consciousness and collective consciousness. *TheScientificWorldJOURNAL* **6,** 1454–1459.
10. Hermansen, T.D., Ventegodt, S., and Merrick, J. (2006). Human development X: explanation of macroevolution — top-down evolution materializes consciousness. The origin of metamorphosis. *TheScientificWorldJOURNAL* **6**, 1656–1666.
11. Hermansen, T.D., Ventegodt, S, Kandel, I., and Merrick, J. (2007) Human development XI: the structure of the cerebral cortex: are there really modules in the brain? *TheScientificWorldJOURNAL* **7,** 1922–1929.
12. Ventegodt, S., Andersen, N.J., and Merrick, J. (2003) Five theories of human existence. *TheScientificWorldJOURNAL* **3**, 1272–1276.
13. Ventegodt, S. (2003) The life mission theory: a theory for a consciousness-based medicine. *Int. J. Adolesc. Med. Health* **15(1)**, 89–91.
14. Ventegodt, S., Andersen, N.J., and Merrick, J. (2003) The life mission theory II. The structure of the life purpose and the ego. *TheScientificWorldJOURNAL* **3**, 1277–1285.
15. Ventegodt, S., Andersen, N.J., and Merrick, J. (2003) The life mission theory III. Theory of talent. *TheScientificWorldJOURNAL* **3**, 1286–1293.
16. Ventegodt, S. and Merrick, J. (2003) The life mission theory IV. A theory of child development. *TheScientificWorldJOURNAL* **3**, 1294–1301.
17. Ventegodt, S., Andersen, N.J., and Merrick, J. (2003) The life mission theory V. A theory of the anti-self and explaining the evil side of man. *TheScientificWorldJOURNAL* **3**, 1302–1313.
18. Ventegodt, S., Andersen, N.J., and Merrick, J. (2003) The life mission theory VI. A theory for the human character: healing with holistic medicine through recovery of character and purpose of life. *TheScientificWorldJOURNAL* **4**, 859–880.
19. Ventegodt, S., Flensborg-Madsen, T., Andersen, N.J., and Merrick J. (2005) The life mission theory VII. Theory of existential (Antonovsky) coherence: a theory of quality of life, health and ability for use in holistic medicine. *TheScientificWorldJOURNAL* **5**, 377–389.
20. Ventegodt, S. and Merrick, J. (2008) Life mission theory VIII: A theory for pain. *J. Pain Manage.,* accepted for publication.
21. Ventegodt, S., Andersen, N.J., and Merrick, J. (2003) Holistic medicine: scientific challenges. *TheScientificWorldJOURNAL* **3**, 1108–1116.
22. Ventegodt, S., Andersen, N.J., and Merrick, J. (2003) The square-curve paradigm for research in alternative, complementary and holistic medicine: a cost-effective, easy and scientifically valid design for evidence based medicine. *TheScientificWorldJOURNAL* **3**, 1117–1127.
23. Ventegodt, S., Andersen, N.J., and Merrick, J. (2003) Holistic medicine III: the holistic process theory of healing. *TheScientificWorldJOURNAL* **3**, 1138–1146
24. Ventegodt, S., Andersen, N.J., and Merrick, J. (2003) Holistic medicine IV: principles of existential holistic group therapy and the holistic process of healing in a group setting. *TheScientificWorldJOURNAL* **3**, 1388–1400.
25. Ventegodt, S. and Merrick J. (2004) Clinical holistic medicine: applied consciousness-based medicine.

*TheScientificWorldJOURNAL* **4**, 96–99.
26. Ventegodt, S., Morad, M., and Merrick, J. (2004) Clinical holistic medicine: classic art of healing or the therapeutic touch. *TheScientificWorldJOURNAL* **4**, 134–147.
27. Ventegodt, S., Morad, M., and Merrick, J. (2004) Clinical holistic medicine: the "new medicine", the multi-paradigmatic physician and the medical record. *TheScientificWorldJOURNAL* **4**, 273–285.
28. Ventegodt, S., Morad, M., and Merrick, J. (2004) Clinical holistic medicine: holistic pelvic examination and holistic treatment of infertility. *TheScientificWorldJOURNAL* **4**, 148–158.
29. Ventegodt, S., Morad, M., Hyam, E., and Merrick, J. (2004) Clinical holistic medicine: use and limitations of the biomedical paradigm *TheScientificWorldJOURNAL* **4**, 295–306.
30. Ventegodt, S., Morad, M., Kandel, I., and Merrick, J. (2004) Clinical holistic medicine: social problems disguised as illness. *TheScientificWorldJOURNAL* **4**, 286–294.
31. Ventegodt, S., Morad, M., Andersen, N.J., and Merrick, J. (2004) Clinical holistic medicine: tools for a medical science based on consciousness. *TheScientificWorldJOURNAL* **4**, 347–361.
32. Ventegodt, S., Morad, M., and Merrick, J. (2004) Clinical holistic medicine: prevention through healthy lifestyle and quality of life. *Oral Health Prev. Dent.* **1**, 239–245.
33. Ventegodt, S., Morad, M., Hyam, E., and Merrick, J. (2004) Clinical holistic medicine: when biomedicine is inadequate. *TheScientificWorldJOURNAL* **4**, 333–346.
34. Ventegodt, S., Morad, M., and Merrick, J. (2004) Clinical holistic medicine: holistic treatment of children. *TheScientificWorldJOURNAL* **4**, 581–588.
35. Ventegodt, S., Morad, M., and Merrick, J. (2004) Clinical holistic medicine: problems in sex and living together. *TheScientificWorldJOURNAL* **4**, 562–570.
36. Ventegodt, S., Morad, M., Hyam, E., and Merrick, J. (2004) Clinical holistic medicine: holistic sexology and treatment of vulvodynia through existential therapy and acceptance through touch. *TheScientificWorldJOURNAL* **4**, 571–580.
37. Ventegodt, S., Flensborg-Madsen, T., Andersen, N.J., Morad, M., and Merrick, J. (2004) Clinical holistic medicine: a pilot study on HIV and quality of life and a suggested treatment of HIV and AIDS. *TheScientificWorldJOURNAL* **4**, 264–272.
38. Ventegodt, S., Morad, M., and Merrick, J. (2004) Clinical holistic medicine: induction of spontaneous remission of cancer by recovery of the human character and the purpose of life (the life mission). *TheScientificWorldJOURNAL* **4**, 362–377.
39. Ventegodt, S., Morad, M., Kandel, I., and Merrick, J. (2004) Clinical holistic medicine: treatment of physical health problems without a known cause, exemplified by hypertension and tinnitus. *TheScientificWorldJOURNAL*.**4**, 716–724.
40. Ventegodt, S., Morad, M., and Merrick, J. (2004) Clinical holistic medicine: developing from asthma, allergy, and eczema. *TheScientificWorldJOURNAL*.**4**, 936–942.
41. Ventegodt, S., Morad, M., Press, J., Merrick, J., and Shek, D.T.L. (2004) Clinical holistic medicine: holistic adolescent medicine. *TheScientificWorldJOURNAL* **4**, 551–561.
42. Ventegodt, S., Solheim, E., Saunte, M.E. Morad, M., Kandel, I., and Merrick, J. (2004) Clinical holistic medicine: metastatic cancer. *TheScientificWorldJOURNAL* **4**, 913–935.
43. Ventegodt, S., Morad, M., Kandel, I., and Merrick, J. (2004) Clinical holistic medicine: a psychological theory of dependency to improve quality of life. *TheScientificWorldJOURNAL* **4**, 638–648.
44. Ventegodt, S. and Merrick, J. (2005) Clinical holistic medicine: chronic infections and autoimmune diseases. *TheScientificWorldJOURNAL* **5**, 155–164.
45. Ventegodt, S., Kandel, I., Neikrug, S., and Merrick, J. (2005) Clinical holistic medicine: holistic treatment of rape and incest traumas. *TheScientificWorldJOURNAL* **5**, 288–297.
46. Ventegodt, S., Morad, M., and Merrick, J. (2004) Clinical holistic medicine: chronic pain in the locomotor system. *TheScientificWorldJOURNAL* **5**,165–172.
47. Ventegodt, S. and Merrick, J. (2005) Clinical holistic medicine: chronic pain in internal organs. *TheScientificWorldJOURNAL* **5**, 205–210
48. Ventegodt, S., Kandel, I., Neikrug, S., and Merrick, J. (2005) Clinical holistic medicine: the existential crisis--life crisis, stress, and burnout. *TheScientificWorldJOURNAL* **5**, 300–312
49. Ventegodt, S., Gringols, G., and Merrick, J. (2005) Clinical holistic medicine: holistic rehabilitation. *TheScientificWorldJOURNAL* **5**, 280–287.
50. Ventegodt, S., Andersen, N.J., Neikrug, S., Kandel, I., and Merrick, J (2005) Clinical holistic medicine: mental disorders in a holistic perspective. *TheScientificWorldJOURNAL* **5**, 313–323.
51. Ventegodt, S., Andersen, N.J., Neikrug, S., Kandel, I., and Merrick, J (2005) Clinical holistic medicine: holistic treatment of mental disorders. *TheScientificWorldJOURNAL* **5**, 427–445
52. Ventegodt, S. and Merrick, J. (2005) Clinical holistic medicine: the patient with multiple diseases *TheScientificWorldJOURNAL* **5**, 324–339.
53. Ventegodt, S., Clausen, B., Nielsen, M.L., and Merrick, J. (2006) Advanced tools for holistic medicine. *TheScientificWorldJOURNAL* **6**, 2048–2065.
54. Ventegodt, S., Clausen, B., and Merrick, J. (2006) Clinical holistic medicine: the case story of Anna. I. Long-term effect of child sexual abuse and incest with a treatment approach. *TheScientificWorldJOURNAL* **6**, 1965–1976.
55. Ventegodt, S., Clausen, B., and Merrick, J. (2006) Clinical holistic medicine: the case story of Anna. II. Patient diary as a tool in treatment. *TheScientificWorldJOURNAL* **6,** 2006–2034.
56. Ventegodt, S., Clausen, B., and Merrick, J. (2006) Clinical holistic medicine: the case story of Anna. III. Rehabilitation of philosophy of life during holistic existential therapy for childhood sexual abuse. *TheScientificWorldJOURNAL* **6**, 2080–2091.
57. Ventegodt, S. and Merrick J. (2005) Suicide from a holistic point of view. *TheScientificWorldJOURNAL.* **5**, 759–766.
58. Ventegodt, S., Clausen, B., Omar, H.A., and Merrick, J. (2006) Clinical holistic medicine: holistic sexology and acupressure through the vagina (Hippocratic pelvic massage). *TheScientificWorldJOURNAL* **6,** 2066–2079.
59. Ventegodt, S., Clausen, B., and Merrick, J. (2006) Clinical holistic medicine: pilot study on the effect of vaginal acupressure (Hippocratic pelvic massage). *TheScientificWorldJOURNAL* **6**, 2100–2116.
60. Ventegodt, S. (2006) Min brug af vaginal akupressur. (My use of acupressure.) *Ugeskr. Laeger* **168(7)**, 715–716. [Danish]
61. Ventegodt, S., Thegler, S., Andreasen, T., Struve, F., Enevoldsen, L., Bassaine, L., Torp, M., and Merrick, J. (2006) Clinical holistic medicine: psychodynamic short-time therapy complemented with bodywork. A clinical follow-up study of 109 patients. *TheScientificWorldJOURNAL* **6**, 2220–2238.
62. Ventegodt, S., Thegler, S., Andreasen, T., Struve, F., Enevoldsen, L., Bassaine, L., Torp, M., and Merrick, J. (2007) Clinical holistic medicine (mindful, short-term psychodynamic psychotherapy complemented with bodywork) in the treatment of experienced impaired sexual functioning. *TheScientificWorldJOURNAL* **7**, 324–329.
63. Ventegodt, S., Thegler, S., Andreasen, T., Struve, F., Enevoldsen, L., Bassaine, L., Torp, M., and Merrick, J. (2007). Clinical holistic medicine (mindful, short-term psychodynamic psychotherapy complemented with bodywork) improves quality of life, health, and ability by induction of Antonovsky salutogenesis. *TheScientificWorldJOURNAL* **7**, 317–323.
64. Ventegodt, S., Thegler, S., Andreasen, T., Struve, F., Enevoldsen, L., Bassaine, L., Torp, M., and Merrick, J. (2007). Clinical holistic medicine (mindful, short-term psychodynamic psychotherapy complemented with bodywork) in the treatment of experienced physical illness and chronic pain. *TheScientificWorldJOURNAL* **7**, 310–316.
65. Ventegodt, S., Thegler, S., Andreasen, T., Struve, F., Enevoldsen, L., Bassaine, L., Torp, M., and Merrick, J. (2007) Clinical holistic medicine (mindful, short-term psychodynamic psychotherapy complemented with bodywork) in the treatment of experienced mental illness. *TheScientificWorldJOURNAL.* **7**, 306–309.
66. Ventegodt, S., Thegler, S., Andreasen, T., Struve, F., Enevoldsen, L., Bassaine, L., Torp, M., and Merrick, J. (2007). Self-reported low self-esteem. Intervention and follow-up in a clinical setting. *TheScientificWorldJOURNAL* **7**, 299–305.
67. Jones, E. (1961) *The Life and Works of Sigmund Freud*. Basic Books, New York.
68. Jung, C.G. (1964) *Man and His Symbols*. Anchor Press, New York.
69. Jung, C.G. (1968). *Psychology and Alchemy. Collected Works of C.G. Jung*. Vol. 12. Princeton University Press, Princeton, NJ.
70. Reich, W. (1969) *Die Function des Orgasmus*. Kiepenheuer Witsch, Köln. [German]
71. Lowen, A. (2004) *Honoring the Body*. Bioenergetics Press, Alachua, FL.
72. Rosen, M. and Brenner, S. (2003) *Rosen Method Bodywork. Accessing the Unconscious Through Touch.* North Atlantic Books, Berkeley.
73. Anand, M. (1989) *The Art of Sexual Ecstasy. The Path of Sacred Sexuality for Western Lovers*. Jeremy P. Tarcher/Putnam, New York.
74. Antonovsky, A. (1985) *Health, Stress and Coping*. Jossey-Bass, London.
75. Antonovsky, A. (1987) *Unravelling the Mystery of Health. How People Manage Stress and Stay Well.* Jossey-Bass, San Francisco.
76. Leichsenring, F., Rabung, S., and Leibing, E. (2004) The efficacy of short-term psychodynamic psychotherapy in specific psychiatric disorders: a meta-analysis. *Arch. Gen. Psychiatry* **61(12),** 1208–1216.
77. Leichsenring, F. (2005) Are psychodynamic and psychoanalytic therapies effective? A review of empirical data. *Int. J. Psychoanal.* **86(Pt 3),** 841–868.
78. Leichsenring, F. and Leibing, E. (2007) Psychodynamic psychotherapy: a systematic review of techniques, indications and empirical evidence. *Psychol. Psychother.* **80(Pt 2),** 217–228.
79. Kelso, J.A.S. (1995) *Dynamic Patterns: The Self-Organization of Brain and Behavior*. MIT Press, London.
80. Kelso, J.A.S. and Engstrom, D.A (2006) *The Complementary Nature*. MIT Press, London.
81. Wolfram, S. (2002) *A New Kind of Science.* Wolfram Media, Champaign, IL.
82. Ventegodt, S. (1999) *Philosophy of Life that Heals. [Livsfilosofi der helbreder.]* Forskningscenterets Forlag, Copenhagen. [Danish]
83. Penrose, R. (1996) *Shadows of the Mind*. Oxford University Press, Oxford.
84. Hofstadter, D.R. (2006) What is it like to be a strange loop? In *Self-Representational Approaches to Consciousnesses.* Kriegel, U. and Williford, K., Eds. MIT Press, London.
85. Grof, S. (1980) *LSD Psychotherapy: Exploring the Frontiers of the Hidden Mind.* Hunter House, Alameda, CA.
86. Grof, S. (2003) Implications of modern consciousness research for psychology: holotropic experiences and their healing and heuristic potential. *Humanistic Psychol.* **31(2–3)**, 50–85.
87. Goleman D. (1997) Healing Emotions: Conversations with the Dalai Lama on the Mindfulness, Emotions, and Health. Mind Life Institute, Boston.
88. Kandel, E.R. and Schwartz, J.H. (2000) *Principles of Neural Science*. Elsevier, Amsterdam.
89. Freeman, W.J. (1967) Analysis of cerebral cortex by use of control systems theory. *Logistics Rev.* **3,** 5–40.
90. Babloyantz, A. (1977) Self-organization phenomena resulting from cell-cell contact. *J. Theor. Biol.* **68(4)**, 551–561.
91. Babloyantz, A. and Kaczmarek, L.K. (1979) Self-organization in biological systems with multiple cellular contacts. *Bull. Math. Biol.* **41(2)**, 193–201.
92. Kohonen, T. (1984) *Selforganization and Associative Memory*. Springer Verlag, Berlin.
93. Lassen, N.A., Roland, P.E., Larsen, B., Melamed, E., and Soh, K. (1977) Mapping of human cerebral functions: a study of the regional cerebral blood flow pattern during rest, its reproducibility and the activations seen during basic sensory and motor functions. *Acta Neurol. Scand. Suppl.* **64**, 262–263, 274–275.
94. Friberg, L., Olsen, T.S., Roland, P.E., Paulson, O.B., and Lassen, N.A. (1985) Focal increase of blood flow in the cerebral cortex of man during vestibular stimulation. *Brain* **8(3)**, 609–623.
95. Szentagothai, J. (1975) The "module concept" in cerebral cortex architecture. *Brain Res.* **95**, 475–496.
96. Mountcastle, V.B. (1978) An organizing principle for cerebral function: the unit module and the distributed system. In *The Mindful Brain.* Edelman, G.M., Ed. MIT Press, Cambridge, MA.
97. Piaget, J. (1954) *The Construction of Reality in the Child*. Basic Books, New York.
98. Piaget, J. (1958) *The Growth of Logical Thinking from Childhood to Adolescence*. Basic Books, New York.
99. Piaget, J. (1969) *The Psychology of the Child*. Basic Books, New York.
100. Campbell, N.A., Reece, J.B., and Mitchell, L.G. (2002) *Biology*. Addison Wesley Longman, Menlo Park, CA.
101. Peitgen, H.-O. (1986) *Beauty of Fractals: Images of Complex Dynamical Systems*. Springer, Berlin.
102. McGinn, C. (2000) *The Mysterious Flame*. Basic Books, New York.

## APPENDIX 1

### THE VALUE OF EXISTING SOURCES OF KNOWLEDGE OF THE BRAIN FOR UNDERSTANDING ITS FUNCTION

#### Sources of Experimental Data about the Structure and Function of the Brain

Data concerning the brain come from different sources, but yet they are not, even when pooled together, adequate to explain fully consciousness and the mind. To postulate that the functions of the brain are understood, we need the collected data to make sense and be able to explain all aspects of the mind, but most of the data does not make much sense, as the EEG and data definitely do not sum up to a nice understanding of what is going on. Some essential sources of data on the brain are mentioned below.

- **Anatomically,** the brain can be divided into forebrain, midbrain, and hindbrain that again are separated into several structures, e.g., cortex cerebri separated into substructures of cerebral cortex. These substructures are split into supracellular structures in the primary visual cortex, meaning analyzers/feature detectors, which seemingly analyze incoming nerve cell signals.
- **The morphogenesis of the brain** is uniform to that of other organs and can be essentially separated from those in reverse order, when axons and dendrites make the connectivity between nerve cells. This connects the different parts of the brain with each other so sensory inputs from receptors in the sense organs and body can be associated with motor outputs of the apparatus of movement.
- **Histological studies**[79] have shown that pyramid cells (that count for approximately half of all cells in the cerebral cortex) converge to about 1,000 of the 3 million cells in the cerebral cortex, positioned only three to four cells apart from each other. Electron microscopic measurements of the distribution of sound impressions in the cortex give the same kinds of results[88,94].
- **Connectivity** — The cortex cerebri can be understood as a surface having a 1,000-dimensional connectivity. The neuron length is about 1,000,000 km, indicating the tremendous complexity of the brain. The existence of meaning-unity analyzers/feature detectors is most evident in the primary visual cortex, but probably exists in all primary cortices. It has been proposed[95,96] that cerebral association-cortex, in general, should be organized in functional modules of 300 μ. However, this is not supported by evidence in the literature because the existing columns only seem to be caused by corticocortical termination from pyramid cells[11].
- **Physiological studies** have shown an intense interaction between all structures of the brain. The thalamus, for instance, may be assumed to play a central role as a regulator of input to the cerebral cortex. Motor outputs that happen through the motor cortex seemingly are essentially influenced by the limbic structures and the basic ganglions, and the patterns of movement (and perceptional patterns) from the cerebellum are also commissioned. Generally, all parts of the brain are mutually connected with each other, but the total brain function is not yet understood.
- **Electrophysiological studies** have shown that inputs are well arranged in the cortex in two-dimensional maps repeated many times throughout the different brain areas. Somatotropic maps in somatosensory and motor areas represent the different sense-receptor types and motor aspects of the body. Visio type maps represent the visual field in the visual areas, and tonotropic maps represent the tone scale. The organization of the smell sense in the cortex is not yet understood. Trials with maps show that these cortical representations can be organized almost instantaneously, and why they hardly are associated directly with axons and dendrites[88].
- **Electron microscopic tests** of complex cells and groups of cells show, most clearly in the primary visual cortex, an organization that is used to analyze meaning-unities of visual impressions.

- **Data from electroencephalograms (EEGs)** are very difficult to interpret. Simultaneously, they reflect the activity from cells in the cortical surface. Normally, the electronic voltage rises and decreases 8–30 times/sec when awake. The most important result is that a high amount of patterns integrates constantly while the brain works[88].
- **Studies of the blood flow in the cerebral cortex**[4,5] and of cortical lesions have given a rather detailed map of the localization of the cerebral cortex's function. We know that data from the primary sensory areas, such as the prefrontal cortex, are dispositioned in areas of higher levels.
- **Psychological studies**[93,94] have shown that the consciousness is built of sense-motor activities and that the development through childhood follows the development of the body's organization. The consciousness consists of sense and motor functions created by sense-motor elements. Later in the development, the child learns to think in abstractions and then to talk. In this way, the consciousness gets freedom to use the body in more complex ways.
- **Philosophy —** What we think gives the consciousness meaning. This interpretation is fundamentally connected with the body. The development of the consciousness as an objective matter of interpretation results in hiding the original physical beginning where the connection between body and mind is obscure.
- **Evolutionary studies** reveal that simple nerve systems, such as *Hydra*, thought to be the first organism with a nerve network, could function through information-transformed interactions between nerve system and health. A quite simple nerve network consisting of neurons, where the nerve impulses can move along in all directions from the stimulus point[100], gives *Hydra* the possibility to carry out very complex behaviors, e.g., catching prey, swimming, and somersaults. Seemingly, this nerve network does not need any practice to function.
- **Mathematical analysis; cortex cerebri as a model machine —** Systems with elements that excitatorically and inhibitorically influence each other, such as neurons do, show a stabile self-organization. However, mathematical analyses show that when the connectivity is huge, as it is in the cortex, the ability to stabilize the self-organization breaks down[90,91], and all patterns become liquid and nondurable.
- **Metabolism —** About 20% of our energy is used by the brain that makes up only 2% of the body's weight. It is estimated that this huge consumption of energy corresponds with an almost constant activity of impulses in all the brain's nerve cells (N.A. Lassen, personal communication, 1990). However, seemingly through an extreme "mental burden", this metabolism does not increase considerably.

### DISCUSSION OF THE SIGNIFICANCE OF THE ABOVE-LISTED DATA SOURCES

How does the brain work? Throughout time, there have been a lot of proposals on how to explain the functions of the brain. However, not one has been able to explain "higher psychic functions" and existing explanations do not seem to support what we know about the brain. For example, it is often proposed that the brain processes its data in the same way as computers. Indeed, computer-implemented "neural networks" with learning-specific "synapse strengths" can be trained to process input patterns into specific output patterns. Coordinated learning through synapse strength is known from lower-level animals[88], but as mentioned, research on the cortical maps have documented that cortical representations are not hard wired. We are, therefore, forced to conclude that the brain does not use "neural networks" of any known type. A neural network is basically a slave without creativity, and does not have an independent activity corresponding to thoughts and dreams, which is another major problem if you try to explain the brain as a neural network. Also, when it comes to consciousness, the nonlocal key quality of consciousness is very difficult to produce even in theory with a neural network; they are popular because they are easy to produce in silicon, but please remember that the electric activity of a computer is extremely local!!! Having dispersed information into a network does not help us to get nonlocal consciousness, as data are now stuck to certain positions in the network. "Convergent groupings of feature detectors" on a number of levels can theoretically give us a single cell that represents any object known from our world, e.g., the famous idea of a "grandmother cell" that fires only when we see our grandmother! However, the problem is, of course, how such activity in a cell should be able to give us the consciousness of seeing our grandmother – how will this single-cell activity be clearly represented in our global brain activity? We believe that models for self-organizing associative memory, such as the famous Kohonen model[92], have the same disability as the other "neural networks" and cannot have an independent activity like thinking. Most interestingly, a truly self-learning neural network has never been constructed; all existing neural networks are in some way controlled from the outside through the programming of the net. Nothing artificial has yet been created that functions like the brain, not even most superficially. This, in itself, should make people think about the difference between machines of silicon and living beings.

The most famous problem in brain research is the psychophysical problem. The materialistic version of this problem goes: How can consciousness emerge from biochemistry? How can any dead bag of inorganic atoms end up feeling and living? Most people intuitively agree that no matter how many balls you put in the bag, or how ingeniously you combine them with electromagnetic springs, they still do not live and laugh. As to the dualistic proposals to find a solution of the psychophysical problem[102], these proposals of the brain function seem to be an expression for philosophic resignation; the problem simply is too hard, so instead of solving it, we cheat. In the end, most researchers who have worked with this problem have actually given up trying to explain consciousness, perception, intention, etc.[102]. Brain research has turned completely mechanical, into molecular research; as if the brain researchers all have come to believe that, just by finding smarter molecules, they will be able to solve the fundamental problems of brain and consciousness. (Maybe that is what is going to happen in the end when we finally get to understand proteins and find "intelligent molecules" that can read the extremely small energies of the directive collective quantum field of the biological system[4,5,6,7,8].)

It is not that we do not believe in science, but we need to acknowledge reality and stop moving away from the fundamental problems that we need to solve; just avoiding the pain of not getting anywhere will not give us a new scientific understanding of the brain. We cannot understand the wholeness as a sum of its parts; the wholeness is always more. That is one of the most central understandings of holistic philosophy[1,2,3,4,5,6,7,8,9,10,11].

The brain (neocortex) makes many extremely complex patterns; that is pretty much what we can tell from all the brain research done thus far. The nature of these patterns; the geometry they must be described in; the way they interact with other patterns, sensory stimuli, and the inner biological informational system of the organism; remains, most unfortunately, still in the dark.
